# Diagnostic accuracy of miRNAs in attention-deficit/hyperactivity disorder: a systematic review and meta-analysis

**DOI:** 10.3389/fpubh.2025.1712404

**Published:** 2025-12-15

**Authors:** Xiaoling Zhong, Hua Yang, Ruling Zhang, Liping Xie, Rong Luo

**Affiliations:** 1 Department of Pediatrics, West China Second University Hospital, Sichuan University, Chengdu, China; 2 Key Laboratory of Obstetric and Gynecologic and Pediatric Diseases and Birth Defects, Ministry of Education, Sichuan University, Chengdu, China; 3 West China Lecheng Hospital, Sichuan University, Qionghai, China

**Keywords:** attention-deficit/hyperactivity disorder, ADHD, miRNAs, biomarkers, diagnosis

## Abstract

**Objective:**

Attention-deficit hyperactivity disorder (ADHD) is a prevalent neurodevelopmental disorder, impacting approximately 5.2% of children globally. The goal of this systematic review and meta-analysis was to evaluate the diagnostic accuracy of miRNAs in identifying ADHD in pediatric and adolescent populations.

**Methods:**

A literature search was conducted in PubMed and CNKI (China National Knowledge Infrastructure) through May 20th, 2025, with no language limitations. Included studies were those reporting the diagnostic accuracy of miRNAs for ADHD in children under 18 years of age. A systematic review and meta-analysis was conducted to determine the combined estimates of the area under the curve (AUC), sensitivity, and specificity for the grouped measurements.

**Results:**

Eleven studies published between 2014 and 2024 reporting on miRNAs were retained. The combined results yielded a sensitivity of 0.82 (0.78–0.86), specificity of 0.82 (0.78–0.85), a positive likelihood ratio (PLR) of 4.45 (3.63–5.47), a negative likelihood ratio (NLR) of 0.22 (0.17–0.27), a diagnostic odds ratio (DOR) of 20.48 (13.92–30.15), and an AUC of 0.89 (0.86–0.91).

**Conclusion:**

The meta-analysis found that miRNAs may serve as diagnostic markers for ADHD in children and adolescents. However, the current included studies remain limited in quantity, with sample sizes likewise constrained. To substantiate this promising potential, future investigations should employ expanded sample cohorts anj01d more rigorously standardized methodologies.

**Systematic review registration:**

https://inplasy.com; unique identifier (INPLASY202570060).

## 1 Introduction

Attention-deficit/hyperactivity disorder (ADHD), a condition commonly manifesting in childhood and the most prevalent neurodevelopmental disorder, is characterized by excessive inattention and/ or hyperactivity-impulsivity, alongside executive dysfunction, poor emotional self-regulation, and diminished motivation (1, 2). Nevertheless, a substantial proportion (65%) of affected children often endure throughout the lifespan and usually comorbid conditions like major depressive disorder (3). The prevalence of ADHD in childhood has been reported to be 5.2% worldwide (4, 5). ADHD is a complex and heterogeneous condition with an incompletely understood etiology, which is recognized as a multifaceted condition arising from interconnected genetic predispositions and environmental risk. While evidence indicates genetic factors studies support a significant contribution, with the average heritability estimate of childhood ADHD being 75% (6).

Current diagnosis of ADHD relies primarily on symptom-based assessment using rating scales, lacking objective and valid biomarkers, which easily leads to misdiagnosis or underdiagnosis, thereby causing delayed treatment or drug misuse. Controversies about the abuse of treatments like methylphenidate have spurred research into objective rather than subjective diagnostic methods of ADHD, though with limited success to date (7, 8). Improving the accuracy of clinical diagnosis is of vital importance, as it ensures that children truly suffering from ADHD can gain timely access to precise treatments. It is generally accepted that objective measures of diagnostic accuracy achieving at least 80% sensitivity and 80% specificity are clinically useful (9). Therefore, the search for objective diagnostic indicators with high diagnostic efficacy is very meaningful for the treatment of children with ADHD.

MicroRNAs (miRNAs) are highly conserved, small non-coding RNAs, usually ranging from 21 to 25 nucleotides in length. They regulate gene expression at the post-transcriptional level by binding to the 3′ UTR of target mRNAs, resulting in either mRNA degradation or translation inhibition (10). MiRNAs are essential in the development of the central nervous system and are involved in various neurological processes, including synaptic plasticity and synaptogenesis (11). Investigating the role of miRNAs in psychiatric disorders could offer valuable insights into the pathogenic mechanisms, which may pave the way for the creation of precise and effective diagnostic and therapeutic approaches. Numerous studies have shown that circulating miRNAs can serve as effective biomarkers for assessing neurodevelopmental disorders (12–14). For instance, abnormal miRNA expression has been identified in patients with schizophrenia and autism spectrum disorders (12, 13).

To date, despite the fact that some meta-analyses have pinpointed variations in a range of biomarkers between children diagnosed with ADHD and their healthy counterparts, there is still a lack of quantitative clarity regarding which diagnostic instruments demonstrate greater precision within particular clinical scenarios (15–17). Some systematic reviews qualitatively summarized various biomarkers, such as scales, neuroimaging, genetic markers, and biochemical indicators, which could serve as potential diagnostic markers for ADHD, whereas, there is a lack of objective quantitative measurement of diagnostic efficiency (18–20). A previous review qualitatively summarized literature on miRNAs as diagnostic markers for ADHD but lacked quantitative analysis of child-specific diagnostic indicators (21). To the best of our knowledge, this is the first systematic review and meta-analysis to quantitatively assess the diagnostic performance of miRNAs in ADHD. The aim of this systematic review and meta-analysis is to quantitatively analyze the diagnostic capability of miRNAs for ADHD, ultimately offering a foundation for future research exploring the underlying mechanisms of ADHD.

## 2 Methods

The protocol for this meta-analysis received prospective registration on the International Prospective Register of Systematic Reviews (INPLASY) with the unique identifier INPLASY202570060. To guarantee methodological rigor and enhance research transparency, the study protocol was designed in strict compliance with the guidelines outlined in the Preferred Reporting Items for Systematic Reviews and Meta-Analyses (PRISMA) statement (22).

### 2.1 Search strategy

**Figure 1:**
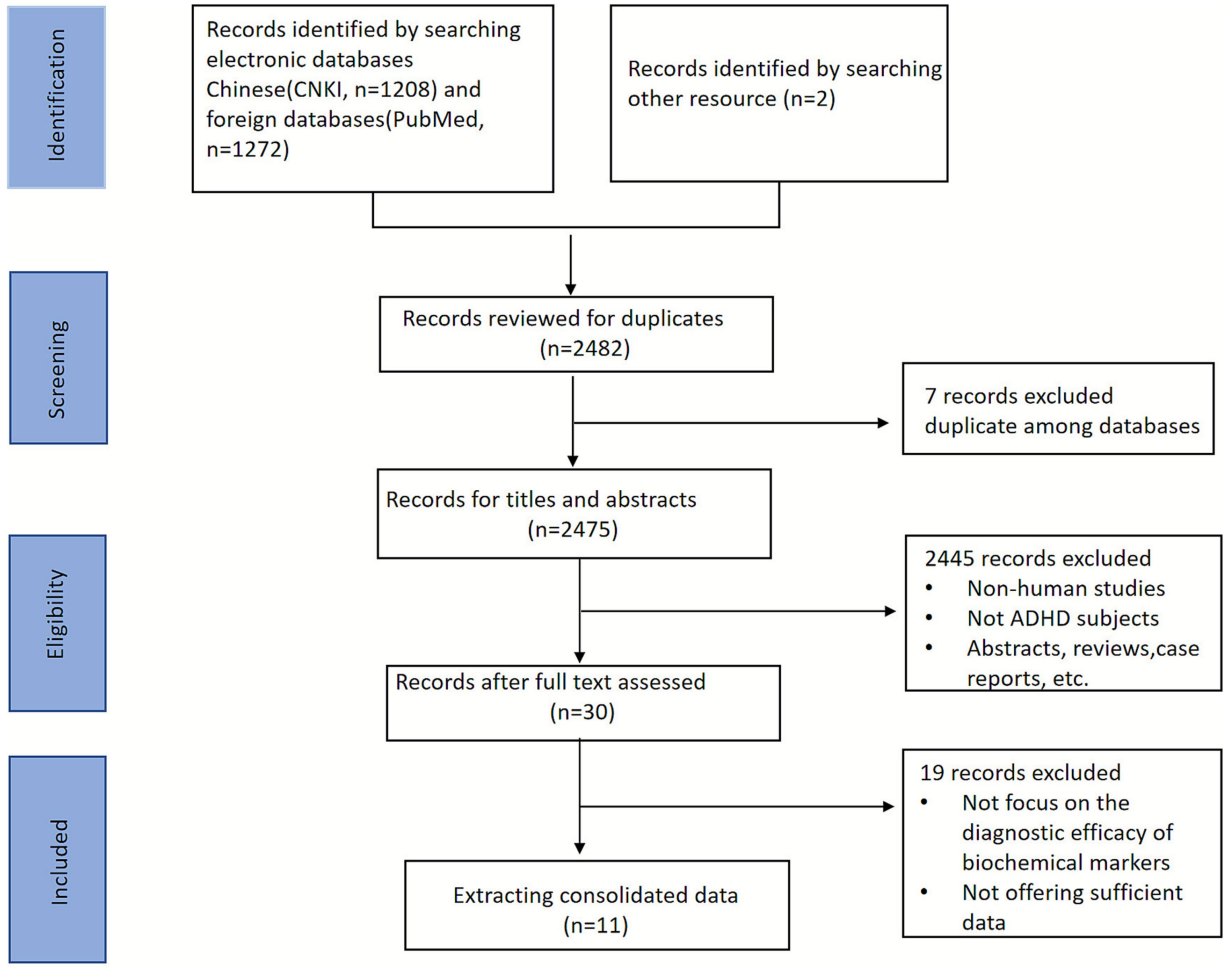
Flowchart of study selection. ADHD, attention deficit/hyperactivity disorder.

A comprehensive literature search was independently conducted across four electronic databases: PubMed and CNKI (China National Knowledge Infrastructure). The search timeframe included all studies published from database inception to May 20, 2025. To reduce publication bias as much as possible, supplementary studies were retrieved by manually screening the reference sections of pertinent articles and searching through gray literature. Relevant keywords were as follows and combined with Boolean operators: (“biomarker” OR “marker” OR “endophenotype”), AND (“ADHD” OR “attention deficit” OR “attention-deficit” OR “attention deficit/ hyperactivity disorder”). A PRISMA diagram illustrating the selection process is presented in Figure 1.

### 2.2 Study selection

Research articles were considered eligible for inclusion if they satisfied the following conditions: (1) Diagnostic accuracy studies that reported the performance of miRNAs with sufficient data for quantitative synthesis (e.g., true positives, false positives, mean differences); (2) Participants: pediatric patients (<18 years) with a confirmed diagnosis of ADHD.

Studies were excluded if: (1) animal study, reviews, editorials, letters, or conference abstracts without original data; (2) Participants were adult patients (≥18 years); (3) Data were insufficient to calculate effect sizes (e.g., incomplete 2 × 2 contingency tables).

### 2.3 Data extraction

After removing duplicates using EndNote25, two reviewers (H Yang and RL Zhang) independently titles and abstracts were evaluated based on the predefined inclusion criteria, and full-text articles of studies deemed potentially relevant were obtained for detailed review. Any inconsistencies during the selection process were addressed through discussion, with unresolved issues adjudicated by a third reviewer (XL Zhong).

Data extraction was performed using a standardized form by the same two reviewers, with cross-verification by a third party. The collected information comprised study details (such as first author, year of publication, country, and sample size), participant demographic data (age, sex, sample size, diagnostic criteria); biomarker details (specimen, name, deregulation); outcome data (sensitivity, specificity, area under the curve (AUC), true positive, false positive, false negative and true negative for diagnostic accuracy).

### 2.4 Quality assessment

The risk of bias was assessed using the Quality Assessment of Diagnostic Accuracy Studies-2 (QUADAS-2) tool, which examines four domains: patient selection, index test, reference standard, and flow and timing (23). Two reviewers (H Yang and RL Zhang) independently conducted the evaluations, and any disagreements were addressed through discussion or, if necessary, resolved by a third reviewer.

### 2.5 Statistical analysis

Data analysis was carried out with Stata (v12.0), RevMan 5.4, and Meta-Disc 1.4. Heterogeneity was tested using the I^2^ statistic and Cochrane’s Q test. I^2^ > 50% suggested substantial heterogeneity, in which case a random-effects model was applied; otherwise, a fixed-effects model was used (24). Pooled estimates for sensitivity, specificity, positive likelihood ratio (PLR), negative likelihood ratio (NLR), and diagnostic odds ratio (DOR) were generated through a bivariate random-effects approach. A summary receiver operating characteristic (SROC) curve was constructed to determine the overall AUC. Deeks’ funnel plot asymmetry test was applied to examine potential publication bias. Sensitivity analysis, subgroup comparisons, and meta-regression analyses were further conducted (when data allowed)—to investigate possible sources of heterogeneity, such as sample type, study design, or population size.

## 3 Results

### 3.1 Study selection

An initial database search identified 2080 relevant articles (1,272 in PubMed, 1,208 in CNKI). To ensure comprehensiveness, 2 additional studies were manually retrieved from the reference lists of prior systematic reviews and meta-analyses. Duplicates were removed using EndNote 25 software, leaving 2,375 articles. Following initial title and abstract screening, 30 studies were included, which were then further screened by full-text review. After a stepwise screening process, 11 articles were ultimately included. The literature screening process and results are depicted in Figure 1.

### 3.2 Characteristics of the included studies

Table 1 provides a summary of the key features of the studies included in the meta-analysis. From 2014 to 2024, eleven eligible publications (25–35) comprising 30 investigations met the inclusion criteria. Collectively, these studies enrolled 2,009 patients diagnosed with ADHD and 2,640 healthy controls. Across the included investigations, sample sizes of ADHD ranged from 9 to 145 participants, whereas control groups varied between 20 and 230 participants. Geographically, the majority were conducted in the China (*n* = 17, including three from Taiwan, China), followed by Iran (*n* = 6), Italy (*n* = 6) and Turkey (*n* = 1). In terms of design, 25 were cross-sectional and 5 were prospective. 7 investigations adopted a multi-center approach, while the remaining 23 were single-center studies. Regarding diagnostic criteria, DSM-5 was applied in 20 studies, DSM-IV in 9, and ICD-10 in only 1. Besides, 19 studies used serum, 1 study used plasma, 3 studies used white blood cell (WBC), and 7 study used whole blood (Table 1).

**Table 1:** The primary characteristics of the included studies.

Study ID	Country	Single-center/multi-center	Study design	Diagnos-is standard	Specimen	Reference gene	RNA	Expression	Control	Case (male/female)	Age of case	Control (male/female)	Age of control
KJ Ding 2023 (25)	China	Single-center	Cross-sectional	DSM-5	Serum	U6	miR-4763-3p	Down	HC	120 (77/43)	8.13 ± 1.09	100 (65/35)	8.90 ± 2.35
GN Shi 2021 (26)	China	Single-center	Prospective	DSM-5	Serum	β-actin	miR-5692b	Up	HC	40 (32/8)	8.34 ± 1.62	60 (49/11)	8.72 ± 1.7
GN Shi 2021 (26)	China	Single-center	Prospective	DSM-5	Serum	β-actin	miR-18a-5p	Down	HC	40 (32/8)	8.34 ± 1.62	60 (49/11)	8.72 ± 1.7
GN Shi 2021 (26)	China	Single-center	Prospective	DSM-5	Serum	β-actin	combination	NR	HC	40 (32/8)	8.34 ± 1.62	60 (49/11)	8.72 ± 1.7
MY Jiang 2022 (27)	China	Single-center	Cross-sectional	DSM-5	Serum	mir-15b	miR-107	Down	HC	37 (31/6)	8.13 ± 3.56	74 (62/12)	8.45 ± 3.77
SF Zhang 2024 (28)	China	Single-center	Cross-sectional	DSM-5	Plasma	U6	miR-126-5p	Up	HC	70 (38/32)	8.77 ± 2.13	70 (34/36)	8.13 ± 1.90
G Liang 2023 (29)	China	Single-center	Prospective	ICD-10	Serum	U6	miR-137	Down	HC	135 (81/54)	7.05 ± 1.15	121 (72/39)	6.89 ± 1.08
LJ Wang 2018 (30)	Taiwan, China	Single-center	Cross-sectional	DSM-IV	WBC	snoRNA RNU44	miR-140-3p miR-27a-3p miR-101-3p miR-150-5p miR-let-7 g-5p miR-30e-5p miR-223-3p miR-142-5p miR-92a-3p miR-486-5p miR-151a-3p miR-151a-5p miR-126-5p	NR	HC	68 (57/11)	9.1 ± 2.2	54 (31/23)	10.0 ± 2.7
LJ Wang 2018 (30)	Taiwan, China	Single-center	Cross-sectional	DSM-IV	WBC	snoRNA RNU44	miR-140-3p miR-27a-3p miR-101-3p miR-150-5p miR-let-7 g-5p miR-30e-5p miR-223-3p miR-142-5p miR-92a-3p miR-486-5p miR-151a-3p miR-151a-5p miR-126-5p	NR	HC	20 (14/6)	8.7 ± 2.2	20 (14/6)	9.2 ± 2.5
LJ Wang 2022 (31)	Taiwan, China	Single-center	Prospective	DSM-5	WBC	snoRNA RNU44	miR-140-3p miR-27a-3p miR-101-3p miR-150-5p miR-let-7 g-5p miR-30e-5p miR-223-3p miR-142-5p miR-92a-3p miR-486-5p miR-151a-3p miR-151a-5p miR-126-5p	NR	HC	145 (111/34)	8.9 ± 2.2	83 (47/36)	9.9 ± 2.6
Hasan Kandemir 2014 (32)	Turkey	Single-center	Cross-sectional	DSM-IV	Whole blood	mixed RNAs generated from the control group	miR107	Down	HC	52 (42/10)	10.09 ± 2.36	52 (36/16)	10.92 ± 2.96
P Zhu 2022 (33)	China	Multi-center	Cross-sectional	DSM-5	Serum	miR-1273 g-3p	miR-4516 miR-6090 miR-4763-3p miR-4281 miR-4466	NR	HC	120	8.16 ± 1.81	200	8.04 ± 1.71
P Zhu 2022 (33)	China	Multi-center	Cross-sectional	DSM-5	Serum	miR-1273 g-3p	miR-4466	Up	HC	120	8.16 ± 1.81	200	8.04 ± 1.71
P Zhu 2022 (33)	China	Multi-center	Cross-sectional	DSM-5	Serum	miR-1273 g-3p	miR-4516	Down	HC	120	8.16 ± 1.81	200	8.04 ± 1.71
P Zhu 2022 (33)	China	Multi-center	Cross-sectional	DSM-5	Serum	miR-1273 g-3p	miR-6090	Down	HC	120	8.16 ± 1.81	200	8.04 ± 1.71
P Zhu 2022 (33)	China	Multi-center	Cross-sectional	DSM-5	Serum	miR-1273 g-3p	miR-4763-3p	Down	HC	120	8.16 ± 1.81	200	8.04 ± 1.71
P Zhu 2022 (33)	China	Multi-center	Cross-sectional	DSM-5	Serum	miR-1273 g-3p	miR-4281	Down	HC	120	8.16 ± 1.81	200	8.04 ± 1.71
P Zhu 2022 (33)	China	Multi-center	Cross-sectional	DSM-5	Serum	miR-1273 g-3p	Combination	NR	HC	132	8.16 ± 1.81	230	8.04 ± 1.71
Zadehbagheri 2019 (34)	Iran	Single-center	Cross-sectional	DSM-IV	Serum	miR-39	hsa-miR-101-3p	Up	HC	56	9.97 ± 1.44	56	10.06 ± 1.35
Zadehbagheri 2019 (34)	Iran	Single-center	Cross-sectional	DSM-IV	Serum	miR-39	hsa-miR-130a-3p	Up	HC	56	9.97 ± 1.44	56	10.06 ± 1.35
Zadehbagheri 2019 (34)	Iran	Single-center	Cross-sectional	DSM-IV	Serum	miR-39	hsa-miR-138-5p	Up	HC	56	9.97 ± 1.44	56	10.06 ± 1.35
Zadehbagheri 2019 (34)	Iran	Single-center	Cross-sectional	DSM-IV	Serum	miR-39	hsa-miR-195-5p	Up	HC	56	9.97 ± 1.44	56	10.06 ± 1.35
Zadehbagheri 2019 (34)	Iran	Single-center	Cross-sectional	DSM-IV	Serum	miR-39	hsa-miR-106b-5p	Down	HC	56	9.97 ± 1.44	56	10.06 ± 1.35
Zadehbagheri 2019 (34)	Iran	Single-center	Cross-sectional	DSM-IV	Serum	miR-39	Combination	NR	HC	56	9.97 ± 1.44	56	10.06 ± 1.35
Nuzziello 2019 (35)	Italy	Single-center	Cross-sectional	DSM-5	Whole blood	miR-191-5p and miR-93-5p	miR-let7b-5p	Up	HC	9 (9/0)	9.78 ± 2.63	20 (14/6)	8.83 ± 3.26
Nuzziello 2019 (35)	Italy	Single-center	Cross-sectional	DSM-5	Whole blood	miR-191-5p and miR-93-5p	miR-148b-3p	Down	HC	9 (9/0)	9.78 ± 2.63	20 (14/6)	8.83 ± 3.26
Nuzziello 2019 (35)	Italy	Single-center	Cross-sectional	DSM-5	Whole blood	miR-191-5p and miR-93-5p	miR-181a-5p	Up	HC	9 (9/0)	9.78 ± 2.63	20 (14/6)	8.83 ± 3.26
Nuzziello 2019 (35)	Italy	Single-center	Cross-sectional	DSM-5	Whole blood	miR-191-5p and miR-93-5p	miR-320a	Up	HC	9 (9/0)	9.78 ± 2.63	20 (14/6)	8.83 ± 3.26
Nuzziello 2019 (35)	Italy	Single-center	Cross-sectional	DSM-5	Whole blood	miR-191-5p and miR-93-5p	miR-652-3p	Up	HC	9 (9/0)	9.78 ± 2.63	20 (14/6)	8.83 ± 3.26
Nuzziello 2019 (35)	Italy	Single-center	Cross-sectional	DSM-5	Whole blood	miR-191-5p and miR-93-5p	miR-942-5p	Up	HC	9 (9/0)	9.78 ± 2.63	20 (14/6)	8.83 ± 3.26

NR, not report.

### 3.3 Diagnostic performance of miRNAs in ADHD

**Figure 2:**
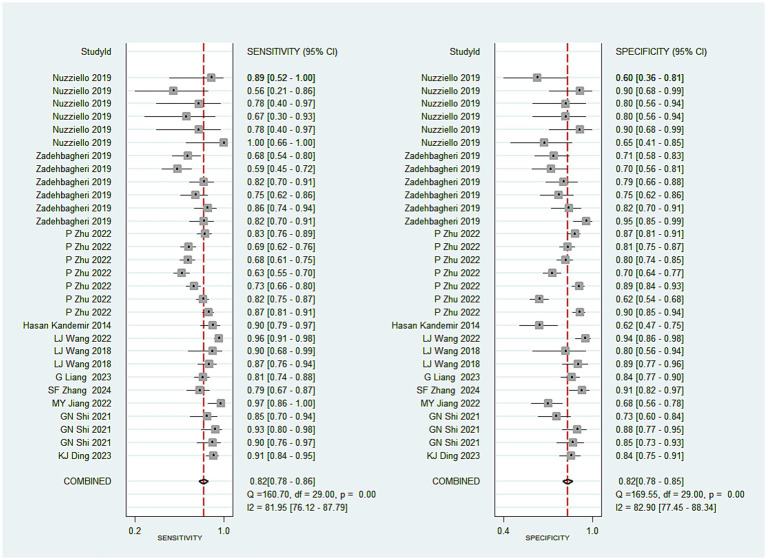
Pooled sensitivity and specificity of miRNAs in ADHD.

**Figure 3:**
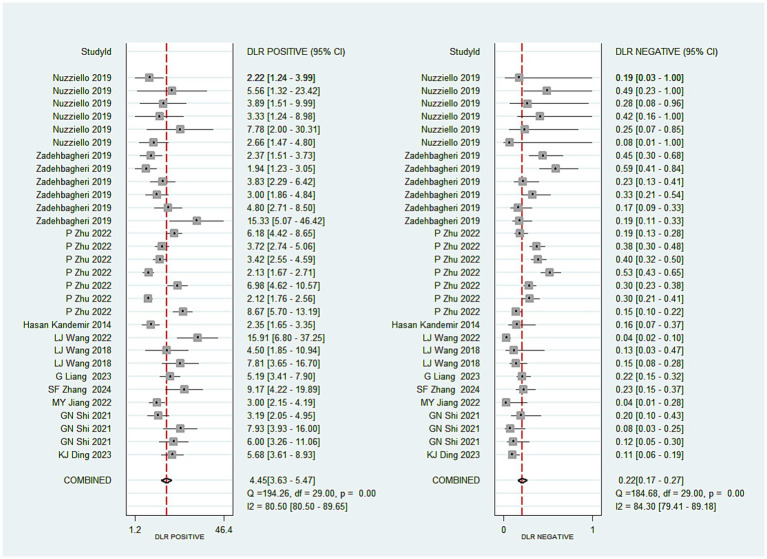
Pooled positive likelihood ratio and negative likelihood ratio of miRNAs in ADHD.

**Figure 4:**
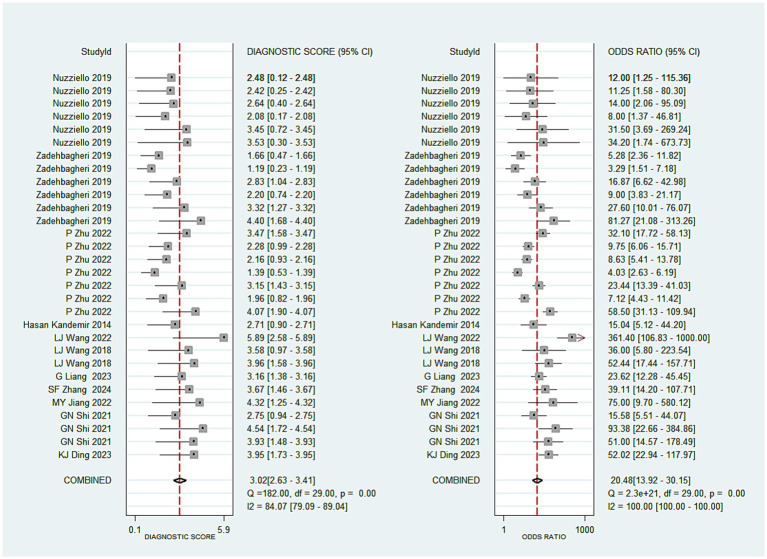
Pooled diagnostic odds ratio of miRNAs in ADHD.

**Figure 5:**
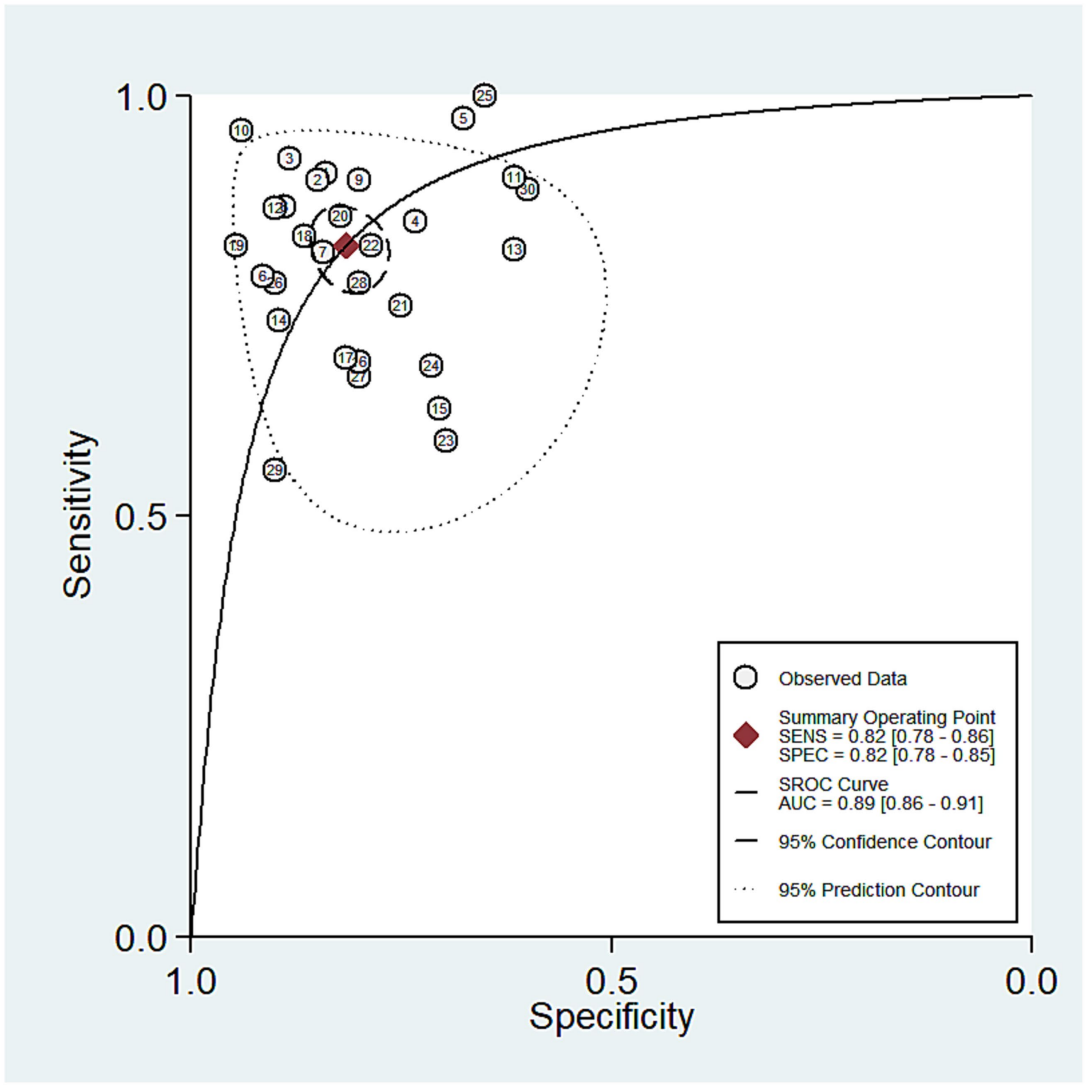
SROC of miRNAs in ADHD.

A total of 30 studies investigated the diagnostic performance of miRNAs for ADHD in children. The analysis revealed substantial heterogeneity (I^2^ = 83.0%, *p* < 0.05), prompting the use of a random-effects model. The aggregated results demonstrated a sensitivity of 0.82 (0.78–0.86), specificity of 0.82 (0.78–0.85), a positive likelihood ratio (PLR) of 4.45 (3.63–5.47), a negative likelihood ratio (NLR) of 0.22 (0.17–0.27), a diagnostic odds ratio (DOR) of 20.48 (13.92–30.15), and an AUC of 0.89 (0.86–0.91) (see Figures 2–5). These results indicated that miRNAs demonstrate relatively high sensitivity and specificity for diagnosing ADHD, as shown in Figure 2. Besides, the results showed that the sensitivity and specificity of miR-4763-3p, miRNA-5692b, miRNA-18a-5p, miR-137, hsa-miR-101-3p, and hsa-miR-130a-3p all exceeded 80%, with hsa-miR-101-3p showing the highest diagnostic efficacy of 95.85% (Table 2).

**Table 2:** Main characteristics of the included studies.

Study ID	Cut-off value	Sensitivity	Specificity	AUC	95%CI	TP	FP	FN	TN	PPV	NPV
KJ Ding 2023 (25)	7.58	91.03	83.75	0.8340	0.7910–0.8920	109	16	11	84	0.8705	0.8861
GN Shi 2021 (26)	8.36	89.00	85.38	0.916	0.8650–0.9530	36	9	4	51	0.8023	0.9209
GN Shi 2021 (26)	8.64	92.52	88.26	0.9490	0.9170–0.9730	37	7	3	53	0.8401	0.9465
GN Shi 2021 (26)	NR	85.08	73.27	0.8530	0.7340–0.9290	34	16	6	44	0.6797	0.8805
MY Jiang 2022 (27)	NR	97.30	67.60	0.6090	NR	36	24	1	50	0.6002	0.9804
SF Zhang 2024 (28)	NR	78.60	91.40	0.8200	0.7400–0.9000	55	6	15	64	0.9014	0.8103
G Liang 2023 (29)	2.132	81.48	83.97	0.8150	0.7630–0.8600	110	19	25	102	0.8501	0.8025
LJ Wang 2018 (30)	NR	86.80	88.90	0.9400	NR	59	6	9	48	0.9078	0.8425
LJ Wang 2018 (30)	NR	90.00	80.00	0.9100	NR	18	4	2	16	0.8182	0.8889
LJ Wang 2022 (31)	NR	96.00	94.20	0.9660	NR	139	5	6	78	0.9666	0.9309
Hasan Kandemir 2014 (32)	0.448	90.40	61.50	无	NR	47	20	5	32	0.7	0.865
P Zhu 2022 (33)	NR	86.67	90.00	0.940	0.9010–0.9660	156	20	24	180	0.8864	0.8824
P Zhu 2022 (33)	NR	0.81	0.62	0.7560	0.6960–0.8090	147	77	33	123	0.6561	0.7863
P Zhu 2022 (33)	NR	73.20	89.30	0.8790	0.8310–0.9180	132	21	48	179	0.8603	0.7873
P Zhu 2022 (33)	NR	62.60	70.50	0.6440	0.5800–0.7050	113	59	67	141	0.6563	0.6768
P Zhu 2022 (33)	NR	68.30	79.90	0.7720	0.7140–0.8240	123	40	57	160	0.7536	0.7369
P Zhu 2022 (33)	NR	68.80	81.70	0.7970	0.7400–0.8460	124	37	56	163	0.7719	0.7442
P Zhu 2022 (33)	NR	83.33	86.36	0.9270	0.8890–0.9560	110	31	22	199	0.7781	0.9003
Zadehbagheri 2019 (34)	NR	82.29	94.64	0.9585	NR	46	3	10	53	0.9338	0.8424
Zadehbagheri 2019 (34)	NR	85.71	82.14	0.9416	NR	48	10	8	46	0.8276	0.8518
Zadehbagheri 2019 (34)	NR	75.00	75.00	0.8326	NR	42	14	14	42	0.75	0.75
Zadehbagheri 2019 (34)	NR	82.14	78.57	0.8555	NR	46	12	10	44	0.7931	0.8148
Zadehbagheri 2019 (34)	NR	58.93	69.64	0.7140	NR	33	17	23	39	0.66	0.629
Zadehbagheri 2019 (34)	NR	67.50	71.40	0.6800	NR	38	16	18	40	0.7024	0.6872
Nuzziello 2019 (35)	NR	94.97	66.73	0.7720	NR	9	0	7	13	0.5623	0.9672
Nuzziello 2019 (35)	NR	77.73	89.66	0.8780	NR	7	2	2	18	0.7718	0.8995
Nuzziello 2019 (35)	NR	69.75	77.84	0.75	NR	6	3	4	16	0.5862	0.8512
Nuzziello 2019 (35)	NR	79.96	77.92	0.778	NR	7	2	4	16	0.6197	0.8963
Nuzziello 2019 (35)	NR	0.5983	0.8891	0.733	NR	5	4	2	18	0.7083	0.831
Nuzziello 2019 (35)	NR	89.05	59.05	0.811	NR	8	1	8	12	0.4946	0.923

AUC, area under the curve; TP, true positive; FP, false positive; FN, false negative; TN, true negative; PPV, positive predictive value; NPV, negative predictive value; NR, not report.

Only two studies (26, 33) have examined the ability of miRNAs to distinguish ADHD status before and after treatment, reporting diagnostic performance of 70.4 and 61.7%. These studies found that, after 3 months of treatment, miRNA levels were closer to those observed in the healthy control group compared to pre-treatment levels. Research on miRNAs for ADHD subtype diagnosis is also limited to one study (33), in which a combined miRNA panel showed high diagnostic accuracy for the inattentive, hyperactive–impulsive, and combined subtypes (AUC = 0.9266, 0.9542, and 0.9782, respectively).

### 3.4 Assessment of bias risk and applicability

**Figure 6:**
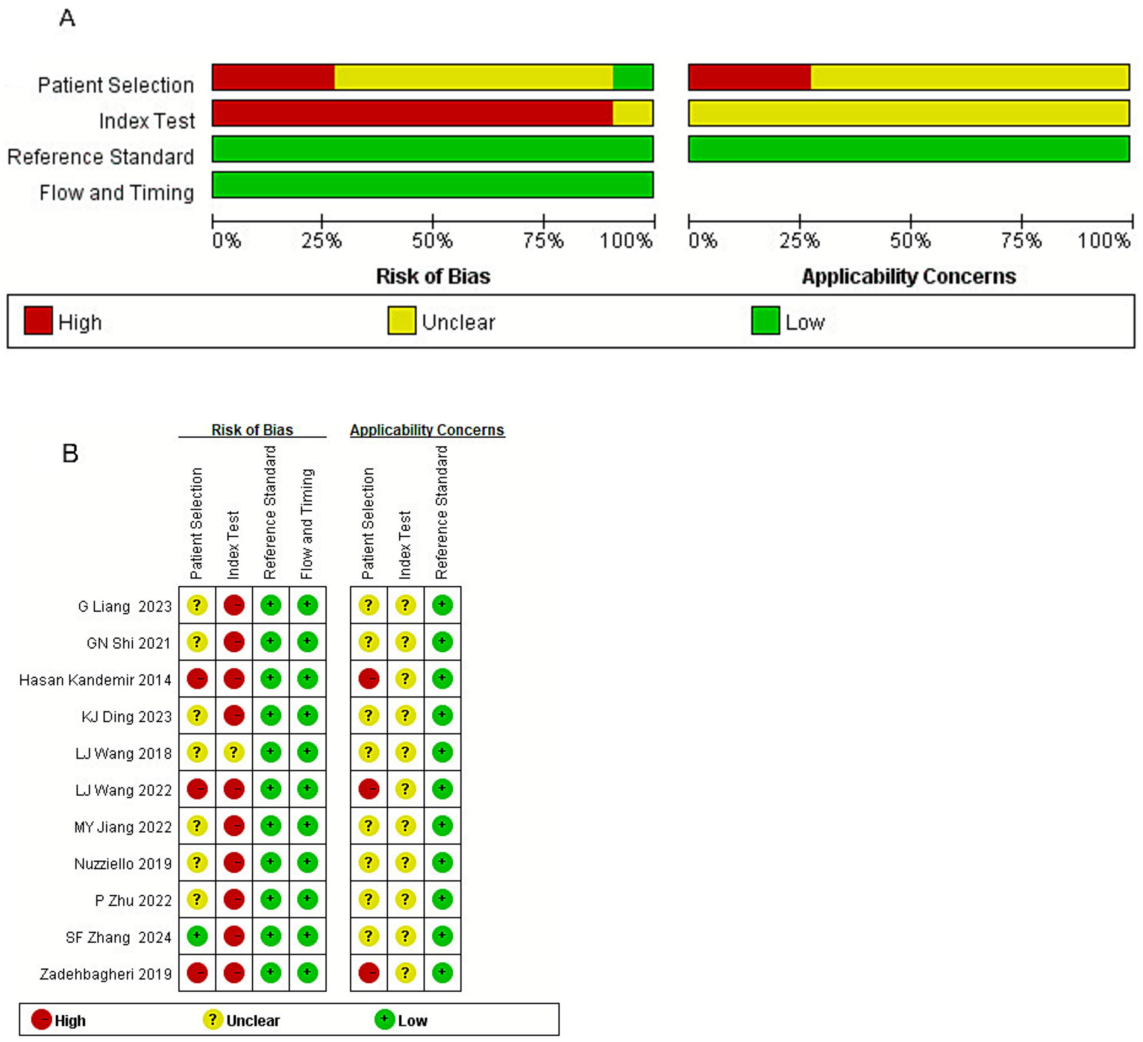
**(A)** Risk of bias and applicability concerns graph: reviews the judgments of the author about each domain presented as percentages across included studies. **(B)** Risk of bias and applicability concerns summary: reviews judgments of the author about each domain for each included study.

A total of eleven investigations were classified as low risk in both the reference standard and flow and timing domains, with low applicability concerns in the reference standard. In contrast, ten studies were deemed high risk in the index test domain, whereas one study revealed unclear risk. Patient selection was predominantly categorized as moderate to high risk, with only one study rated as low risk. Overall, patient selection and index test domains raised moderate to high applicability concerns. In conclusion, the studies incorporated in this review demonstrated a low risk of bias regarding the reference standard as well as flow and timing. However, the patient selection risk was moderate to high, as these studies focused on patients with already established diagnosis. The index test risk was also moderate to high, primarily due to threshold issues and the fact that the tests were conducted with patients whose diagnosis were already known. The results are shown in Figures 6A,B.

### 3.5 Sensitivity, subgroup and meta-regression analysis

We conducted a subgroup analysis based on miRNA profiling, specimen type, and study design. The results indicated that the overall diagnostic performance of multiple miRNAs (AUC = 0.9225 ± 0.0292) was higher than that of individual miRNAs (AUC = 0.8747 ± 0.0166). Additionally, studies with a prospective design showed better diagnostic performance (AUC = 0.9376 ± 0.0421) compared to cross-sectional studies (AUC = 0.8745 ± 0.0163). Regarding specimen types, we found that only one study was based on plasma and whole blood, while the majority of studies (19/30) used serum. The diagnostic performance for studies based on WBC samples (AUC = 0.9605 ± 0.0371) was found to be higher than that of studies using serum samples (AUC = 0.8787 ± 0.0183) and whole blood (AUC = 0.8580 ± 0.0314). The results are displayed in Table 3.

**Table 3:** Subgroup analysis for the selected studies.

Subgroup analysis	Sensitivity (95% CI)	Specificity (95% CI)	PLR (95% CI)	NLR (95% CI)	DOR (95% CI)	AUC
LncRNA profiling						
Single miRNA	0.77 (0.75–0.79)	0.78 (0.77–0.80)	3.81 (3.09–4.71)	0.27 (0.22–0.33)	16.88 (11.31–25.19)	0.8747 ± 0.0166
Multiple miRNAs	0.86 (0.84–0.89)	0.86 (0.83–0.89)	5.60 (3.47–9.05)	0.16 (0.10–0.27)	36.11 (14.77–88.30)	0.9225 ± 0.0292
Study design						
Prospective	0.89 (0.86–0.92)	0.85 (0.81–0.89)	6.14 (3.71–10.16)	0.12 (0.06–0.24)	54.03 (18.72–155.98)	0.9376 ± 0.0421
Cross-sectional	0.77 (0.75–0.79)	0.80 (0.78–0.81)	3.89 (3.13–4.85)	0.27 (0.21–0.33)	16.94 (11.30–25.39)	0.8745 ± 0.0163
Specimen						
Serum	0.78 (0.76–0.79)	0.80 (0.78–0.82)	4.08 (3.21–5.17)	0.25 (0.20–0.32)	17.72 (11.35–27.66)	0.8787 ± 0.0183
Plasma	0.79	0.91	–	–	–	0.8200
WBC	0.93 (0.89–0.96)	0.90 (0.85–0.95)	8.29 (3.97–17.29)	0.09 (0.04–0.22)	94.24 (22.46–395.39)	0.9605 ± 0.0371
Whole blood	0.84 (0.76–0.90)	0.73 (0.65–0.79)	2.67 (2.10–3.41)	0.29 (0.19–0.45)	14.46 (7.41–28.22)	0.8580 ± 0.0214

CI, confidence intervals; PLR, positive likelihood ratio; NLR, negative likelihood ratio; DOR, diagnostic odds ratio; WBC, white blood cell; NR, not report.

To further explore the sources of significant heterogeneity, meta-regression analysis was conducted considering factors such as specimen, sample size and study design. The analysis revealed that specimen did not significantly influence the homogeneity of the meta-analysis (*p* = 0.326, *t* = 1.00, 95% CI −0.1200, 0.3469). Likewise, sample size was not a significant contributing factor (*p* = 0.480, *t* = −0.72, 95% CI −0.542, 0.262). Additionally, the study design had minimal effect on the overall homogeneity (*p* = 0.136, *t* = 1.54, 95% CI −0.130, 0.908). Therefore, none of these factors-specimen, study design and sample size-appeared to account for the observed heterogeneity based on meta-regression analysis.

**Figure 7:**
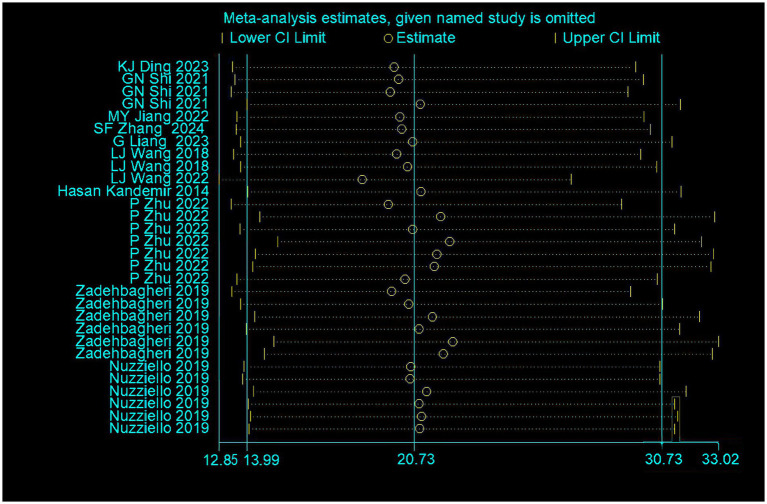
Sensitivity analysis of the meta-analysis.

The sensitivity analysis performed on the meta-pooled results for all miRNAs showed stable outcomes even when individual studies were omitted. A comprehensive overview of the meta-pooled results and the corresponding sensitivity analyses for these biomarkers can be found in Figure 7.

### 3.6 Publication bias

**Figure 8:**
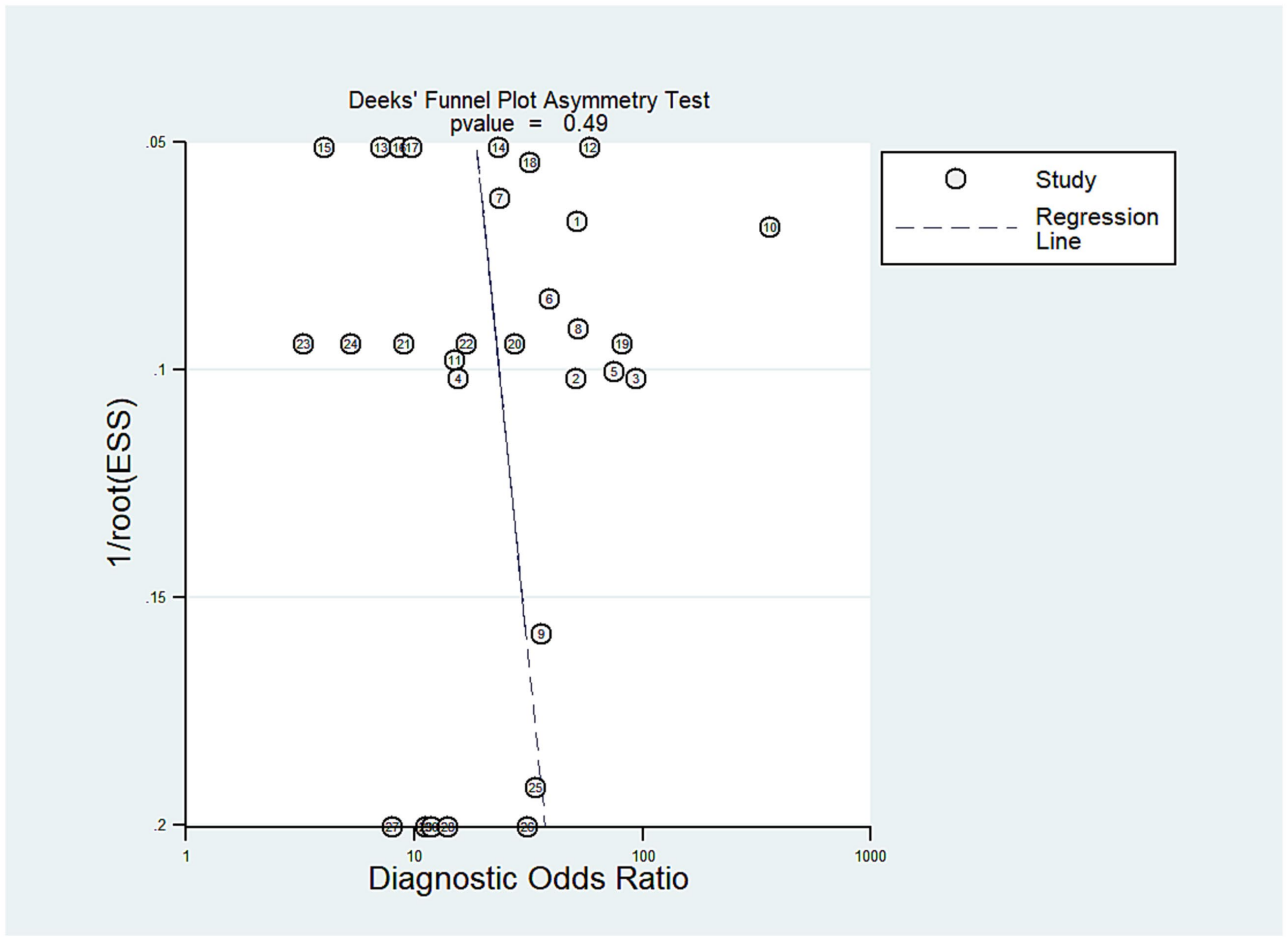
Deek’s funnel plot evaluating the potential publication bias of the included studies. ESS, effective sample size.

As illustrated in Figure 8, Deek’s funnel plot asymmetry test was employed to evaluate potential publication bias, with results indicating no statistically significant bias (*p* > 0.05).

## 4 Discussion

To the best of our knowledge, this is the first meta-analysis providing a quantitative assessment of miRNAs’ diagnostic efficacy in ADHD. Our results showed that the pooled sensitivity and specificity of miRNAs exceed 80%, with a pooled AUC of 0.89, indicating the potential of miRNAs as novel objective diagnostic biomarkers for ADHD. This meta-analysis identified several miRNAs with relatively high diagnostic efficacy, with both sensitivity and specificity exceeding 80%, such as miR-4763-3p, miRNA-5692b, miRNA-18a-5p, miR-137, hsa-miR-101-3p, and hsa-miR-130a-3p, as well as some miRNAs that have been repeatedly investigated across different studies, including miR-107 and miR-4763-3p.

Some studies have explored the potential roles of these miRNAs in ADHD (13, 36–40). The diagnostic efficacy of miR-107 in ADHD was found to be consistent across two studies (27, 32). Previous studies have revealed changes in the expression levels of hsa-miR101-3p, hsa-miR-130a-3p, and hsa-miR-195-5p in relation to autism spectrum disorder (13, 37). Hsa-miR-130a influences neurite growth and dendritic density by downregulating the MECP2 gene, whose dysfunction has been linked to autism spectrum disorder (38). This suggested that shared molecular pathways between ADHD and autism spectrum disorder. The possible involvement of these miRNAs in various neuropsychiatric conditions is compelling, as researchers propose that disorders such as ADHD, autism spectrum disorder, schizophrenia, and anxiety share overlapping neurological characteristics and a common genetic foundation (39). Besides, a previous study suggested that elevated miR-5692b levels contribute to ADHD by targeting and regulating the protein Homer 1a (40). Furthermore, multiple studies have shown that microRNAs (miRNAs) are promising molecular biomarkers that not only contribute to the early diagnosis of various diseases, but also reveal underlying pathogenic mechanisms through their altered expression levels. This has been demonstrated in conditions such as bladder cancer and neurodegenerative diseases(e.g., Alzheimer’s disease and Parkinson’s disease) (41, 42).

Our results indicated that the diagnostic efficacy of combined miRNAs is higher than that of individual miRNAs, which is consistent with a previous study (18). Therefore, future research was supposed to focus on combining miRNAs for more comprehensive analysis. Besides, the results also indicated that studies with a prospective design have a higher AUC compared to those with a cross-sectional design, suggesting that prospective designs may be a better approach for future studies. Furthermore, the included studies involved various specimens, including serum, WBC, whole blood, and plasma. The pooled AUC derived from studies utilizing WBC specimen is higher than that from those employing serum. However, the constrained quantity of investigations encompassed in this meta-analysis, caution is needed when interpreting this condition. Additionally, all samples in the included studies required peripheral blood collection. Future research may benefit from considering alternative bodily fluids, such as saliva or urine.

This meta-analysis does have several limitations. First, although a range of statistical approaches, including sensitivity, subgroup, and meta-regression analyses, were initially implemented, we did not reveal the source of the observed heterogeneity. Despite this, it is plausible that clinical heterogeneity exists, potentially arising from factors such as the use of different internal references, variations in different countries or populations, and differences in study designs, all of which may contribute to clinical variability. Second, the included studies were mainly conducted in Asian populations (Mainland in China, Taiwan in China, Iran, and Turkey), which means geographic limitation could affect the external validity and generalizability to other populations. Third, all studies included in the analysis involved pre-diagnosed patients, and the sample sizes were generally small (<100). Only two studies from China had exceed 100 participants. This raises concerns regarding the quality and reliability of the studies included in the meta-analysis. While such limitations existed, this systematic review and meta-analysis represented the first quantitative evaluation of miRNAs’ diagnostic utility in pediatric ADHD populations. It indicated that miRNAs have the potential to act as novel, promising diagnostic biomarkers for ADHD-providing valuable insights and laying out directions for subsequent research efforts in this domain.

## 5 Conclusion

In summary, this is the first and most comprehensive systematic review and meta-analysis focused on the quantitative evaluation of the diagnostic performance of miRNAs for ADHD in children and adolescents, and is poised to inform future research in this field. Our findings indicated that miRNAs exhibit relatively high diagnostic efficacy in ADHD, particularly when tested in combination of miRNAs; however, these results are constrained by small sample sizes, homogeneous study populations, and limitations in study design. Moving forward, there is a need for research that replicable, standardization, and multimodal approaches, alongside multicenter investigations to enhance the generalizability of findings. Besides, future studies should further investigate miRNAs in subtype differentiation and treatment response evaluation. Research efforts also ought to concentrate on machine learning and multivariate, multi-tiered biomarker strategies-approaches that can reasonably be considered most appropriate for addressing the intricate nature of mental disorders.

## Data Availability

The original contributions presented in the study are included in the article/supplementary material, further inquiries can be directed to the corresponding author.
